# Sequence-Specific Free Energy Changes in DNA/RNA Induced by a Single LNA-T Modification in Antisense Oligonucleotides

**DOI:** 10.3390/ijms252413240

**Published:** 2024-12-10

**Authors:** Elisa Tomita-Sudo, Tomoka Akita, Nae Sakimoto, Saori Tahara-Takamine, Junji Kawakami

**Affiliations:** 1 Konan Laboratory for Oligonucleotide Therapeutics (KOLOT), 7-1-20 Minatojima-Minamimachi, Kobe 650-0047, Japan; 2 Faculty of Frontiers of Innovative Research in Science and Technology (FIRST), Konan University, 7-1-20 Minatojima-Minamimachi, Kobe 650-0047, Japan

**Keywords:** locked nucleic acid, thermodynamic stability, LNA-containing antisense oligonucleotides

## Abstract

2′,4′-methylene bridged nucleic acid/locked nucleic acid (2′,4′-BNA/LNA; LNA) is a modified nucleic acid that improves the function of antisense oligonucleotide therapeutics. In particular, LNA in the DNA strand increases its binding affinity for the target RNA. Predicting the binding affinities of LNA-containing antisense oligonucleotides and RNA duplexes is useful for designing antisense oligonucleotides. The nearest neighbor parameters may be useful for binding affinity prediction, similar to those for natural nucleic acids. However, the sequence dependence of the thermodynamic stability of DNA/RNA duplexes containing LNA remains unexplored. Therefore, in this study, we evaluated the thermodynamic stabilities of DNA/RNA duplexes containing a single LNA modification in the DNA strand. We found that LNA-stabilized DNA/RNA duplexes averaged −1.5 kcal mol^−1^. Our findings suggest that the thermodynamic stabilization effect of LNA is sequence-specific.

## 1. Introduction

Numerous modified nucleic acids have been developed to improve the functionality of antisense therapeutics. Since the approval of fomivirsen for the treatment of retinitis in the United States in 1998, 12 antisense drugs have been approved. 2′-*O*-methyl [1], 2′-*O*-methoxyethyl (2′-MOE) [2], and 2′-fluoro (2′-F) [3] are used to confer nuclease resistance on DNA strands and improve binding affinity to the target RNA. 2′,4′-methylene bridged nucleic acid/locked nucleic acid (2′,4′-BNA/LNA; hereafter referred to as LNA), which was developed by Obika et al. and Wengel et al. [4,5], provides not only nuclease resistance [6] but also superior binding affinity compared to other modified nucleotides developed thus far [7,8].

There are two basic approaches to gene regulation through antisense oligonucleotides (ASOs): one involves target RNA cleavage via RNase H [9,10,11] and the other is based on the inhibition of protein binding to the target RNA during pre-mRNA splicing to induce exon skipping or inclusion [12,13,14]. In both these approaches, efficient gene regulation is achieved if the ASO binds tightly to the target RNA. However, efficient gene regulation may be impaired if the binding affinity between the target mRNA and the ASO is excessively high. In the case of RNase H-dependent ASOs, very stable binding prevents the ability of an ASO to mediate multiple turnovers of target molecules [15,16]. Yamamoto et al. showed that ASOs with a melting temperature (*T*_m_) of 40–60 °C efficiently elicit multiple rounds of RNA cleavage [15]. Pedersen et al.’s work [16] revealed that sequence-specific ASO design is necessary for efficiently regulating gene expression, as each target sequence has an optimal binding affinity for the complementary strand. Their study shows that ASO length, G/C content, and the extent of LNA modification affect genetic repression efficiency, and all of these factors are determinants of the duplex stability. Similarly, a report on the treatment of Duchenne muscular dystrophy via exon skipping found that extremely high binding affinity reduced the activity of splice-switching oligonucleotides containing LNA residues [17]. Moreover, stronger target binding can lead to higher off-target effects. Thus, guidelines for designing molecular ASOs with optimal binding affinities for the target mRNA are necessary. Predicting the duplex stability of LNA-modified ASOs and target RNAs could help predict the function of ASOs. Usually, *T*_m_ and thermodynamic parameters (Gibbs free energy Δ*G*°, enthalpy change Δ*H*°, and entropy change Δ*S*°) have been used to evaluate duplex stability.

In 1998, Singh et al. reported that introducing LNA modifications into DNA duplexes improved *T*_m_ [5]. Since then, similar studies have used *T*_m_ increments to assess functional improvement due to LNA introduction [7,8]. However, *T*_m_ depends on the concentration of oligonucleotides, and there is no theoretical basis for the direct proportional relationship between the *T*_m_ change (Δ*T*_m_) and the number of modifications. Thus, Δ*T*_m_/modification is an inappropriate parameter for the thermodynamic prediction of duplex stability. In contrast, Δ*G*° during duplex formation at 37 °C (Δ*G*°_37_), an energetic parameter of duplex stability, may be suited for predicting duplex stability.

For natural nucleic acids, duplex stability can be predicted using the nearest neighbor (NN) model. This model allows for the estimation of Δ*G*°_37_ from the combination of two adjacent base pairs [18,19,20]. If this model can be applied to duplexes containing LNA, then it would be possible to predict the binding affinity of LNA-modified oligonucleotide therapeutics to target sequences. Several reports have suggested the adaptive potential of the NN model to LNA-modified duplexes. McTigue et al. introduced a single LNA residue into various DNA/DNA duplexes and compared its stabilizing effects [21], suggesting that the thermodynamic stabilization effect differed depending on the base adjacent to the LNA. For RNA/RNA duplexes, Kierzek et al. characterized the stabilization effect of LNA-substitution on the RNA duplexes with 2′-*O*-methyl modification and showed that the stabilizing effect varies depending on the adjacent bases [22].

For ASO therapeutics, the thermodynamic properties of the heteroduplex of LNA-containing DNA and the target RNA should be considered. Kaur et al. [23] stated, “... the melting of an A-type DNA•RNA heteroduplex will release a higher number of water molecules than the melting of a DNA•DNA duplex”. This may suggest that there are different stabilization mechanisms introduced by LNA between DNA/RNA and DNA/DNA duplexes. However, the thermodynamic parameters of DNA/RNA duplexes with LNA modifications have not been well characterized [23], and the parameters are vast. When an LNA residue (N_2_(L)) located immediately 3′ of a natural nucleotide (N_1_) within a sequence is denoted as N_1_N_2_(L), and a natural nucleoside (N_2_) immediately 3′ of an LNA nucleoside (N_1_(L)) as N_1_(L)N_2_, there are 16 possible NN base pair combinations each for N_1_N_2_(L) and N_1_(L)N_2_. Therefore, to verify the applicability of the LNA-containing NN model to DNA/RNA, it is necessary to obtain 32 different NN parameters, even with only one LNA-containing NN pattern. So far, such data are unavailable. However, by testing whether the thermodynamic effect of introducing LNA into the DNA strand of a DNA/RNA duplex on duplex stability is sequence-dependent, we can verify whether duplexes formed with LNA-containing DNA/RNA apply to the NN model.

In this study, we first examined the applicability of the NN model to DNA (with an LNA modification)/RNA duplexes. In detail, we evaluated the thermodynamic parameter changes (Δ*G*°_37_, Δ*H*°, and Δ*S*°) during the formation of 12 DNA/RNA duplexes, with an LNA nucleoside located between two natural nucleosides (N_1_N_2_(L)N_3_), and compared the thermodynamic parameters of duplexes with the same N_1_N_2_(L)N_3_ triplets at another internal position of the duplex. If the thermodynamic parameters of the same N_1_N_2_(L)N_3_ triplets have similar numerical values, then the NN model can be applied to the LNA-containing DNA/RNA duplexes.

## 2. Results

### 2.1. Two State Transitions of DNA/RNA Duplexes

**Figure 1:**
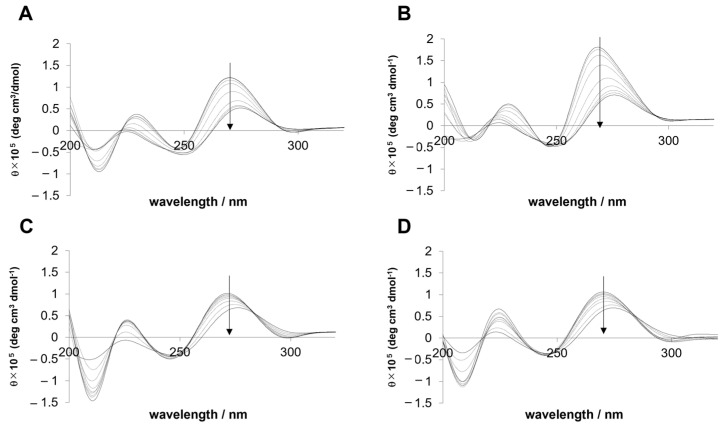
CD spectra of (**A**) i, (**B**) ii, (**C**) iii, and (**D**) iv DNA/RNA duplexes. The total concentration of the oligonucleotides (*C*_t_) was 30 μM. The direction of the arrow indicates the temperature change from 20 °C to 60 °C.

Four antisense DNAs—i(DNA), ii(DNA), iii(DNA), and iv(DNA)—and their complementary RNAs—i(RNA), ii(RNA), iii(RNA), and iv(RNA)—were used in this study. The sequences are provided in the Materials and Methods section. The CD spectra of i(DNA/RNA), ii(DNA/RNA), iii(DNA/RNA), and iv(DNA/RNA) are shown in Figure 1. All spectra exhibited positive peaks at approximately 230 and 270 nm and negative peaks at approximately 210 and 250 nm. The spectral features determined in this study are consistent with the recognized intermediate characteristics between the B-form (DNA/DNA duplexes) and A-form (RNA/RNA duplexes) of the DNA/RNA duplexes [20]. The CD spectra obtained at different temperatures for all the duplexes exhibited isodichroic points, confirming the two-state melting behavior. Additionally, the two-state transition behavior was also supported by the observation that the errors in the parameters obtained from the log*C*_t_ plot and curve fitting were within 10% of each other.

### 2.2. Thermodynamic Parameters from Melting Experiments

The thermodynamic parameters obtained from the curve fitting analysis are presented in Table S1. Δ*H*°, Δ*S*°, and Δ*G*°_37_ were treated as independent values relative to the oligonucleotide concentration, and their average values were used as the parameters obtained from curve fitting (Table S1: curve fitting). The log*C*_t_ plots are shown in Figures S1–S4, and the parameters from log*C*_t_ plots are also listed in Table S1. The errors for all the parameters obtained from each analytical method were less than 10%. The mean parameter values were calculated as the average values of those obtained from curve fitting and log*C*_t_ plots. The resulting thermodynamic parameters of i(DNA/RNA), ii(DNA/RNA), iii(DNA/RNA), and iv(DNA/RNA) are presented in Table 1. The errors for Δ*G*°_37_, Δ*H*°, Δ*S*°, and −*T*Δ*S*° were ±0.0–0.2 kcal mol^−1^, ±1.4–7.0 kcal mol^−1^, ±4.5–20.2 cal mol^−1^ K^−1^, and ±1.4–6.3 kcal mol^−1^, respectively.

Among the natural i–iii series, the iii DNA/RNA duplex was enthalpically the most stable (−89.9 kcal mol^−1^), whereas ii duplex formation was entropically the most favorable (−227.0 cal mol^−1^ K^−1^). Overall, the iii duplex with a higher GC content (50%) than the other duplexes (25%) was the most stable in terms of Δ*G*°_37_ (−12.1 kcal mol^−1^) and *T*_m_ at *C*_t_ = 30 μM (54.7 °C).

**Figure 2:**
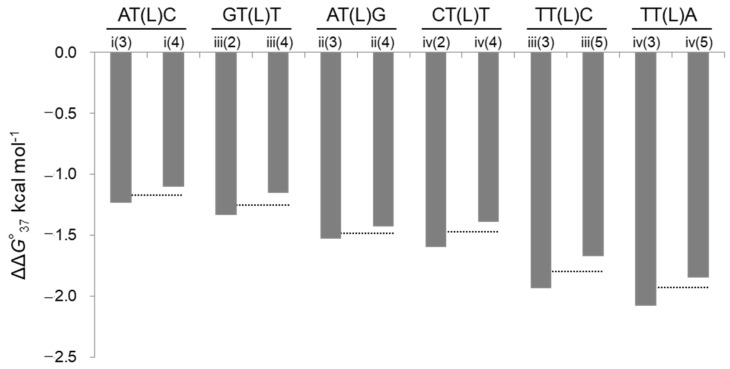
ΔΔ*G*°_37_ values of i–iv series duplexes. The dotted line indicates the average ΔΔ*G*°_37_ for duplexes containing the same N_1_N_2_(L)N_3_ triplet.

The stabilizing effect of LNA was evaluated as ΔΔ*G*°_37_ calculated by subtracting Δ*G*°_37_ for the natural DNA/RNA duplex formation from Δ*G*°_37_ for the LNA-containing duplex formation. Negative values of ΔΔ*G*°_37_ indicate duplex stabilization by LNA introduction. Enthalpic effects, entropic effects, and *T*_m_ changes were similarly quantified as ΔΔ*H*°, −*T*ΔΔ*S*°, and Δ*T*_m_, respectively. ΔΔ*G*°_37_ ranged from −2.0 to −1.1 kcal mol^−1^, indicating that all the LNA-containing DNA/RNA duplexes were thermodynamically stabilized compared to natural duplexes (Table 1 and Figure 2). Similarly, Δ*T*_m_ ranged from 3.9 to 6.9 °C (average 5.4 °C), and the positive Δ*T*_m_ effect also showed that the binding affinity was improved by the introduction of LNA in all cases, as indicated by ΔΔ*G*°_37_.

Additionally, ΔΔ*H*° and −*T*ΔΔ*S*°, which serve as parameters for estimating the stabilization mechanism, varied from negative values (indicating stabilization) to positive values (indicating destabilization) depending on the sequences. ΔΔ*H*° values for i(3), i(4), and ii(3) were positive, while the −*T*ΔΔ*S*° values were negative, indicating entropic stabilization. For the other nine sequences, ΔΔ*H*° was negative, and −*T*ΔΔ*S*° was positive, indicating enthalpic stabilization.

**Figure 3:**
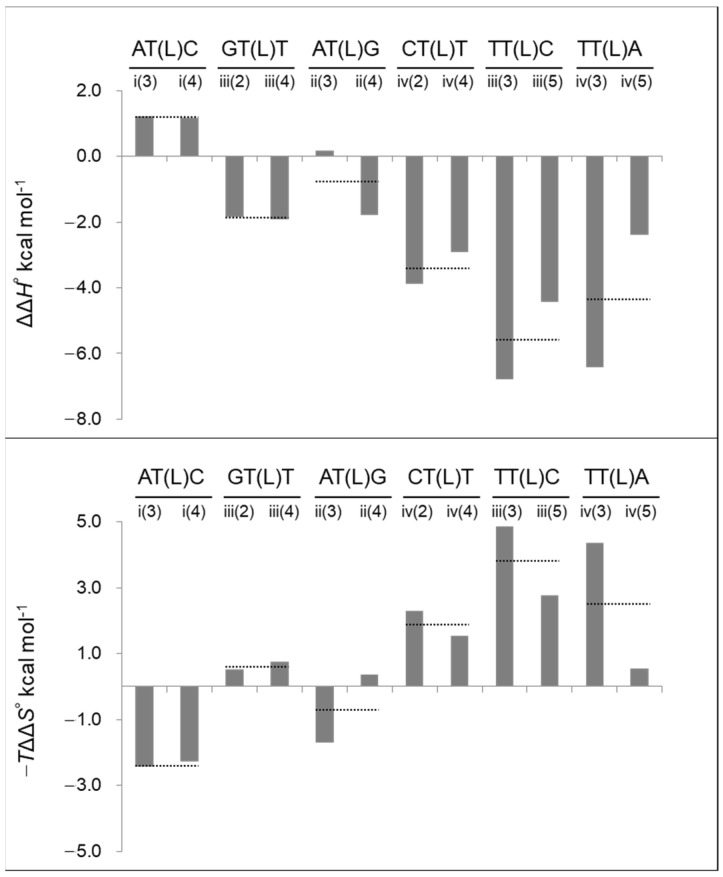
ΔΔ*H*° (upper panel) and −*T*ΔΔ*S*° (lower panel) values of i–iv series duplexes. The dotted line indicates the average ΔΔ*H*° or −*T*ΔΔ*S*° for duplexes containing the same N_1_N_2_(L)N_3_ triplet.

The thermodynamic parameters for duplex formation were compared across several sequences with the same LNA-containing triplet (N_1_N_2_(L)N_3_) (six combinations: i(3) and i(4) (AT(L)C), ii(3) and ii(4) (AT(L)G), iii(2) and iii(4) (GT(L)T), iii(3) and iii(5) (TT(L)C), iv(2) and iv(4) (CT(L)T), and iv(3) and iv(5) (TT(L)A)). As presented in Table 1 and shown in Figure 2, the difference in ΔΔ*G*°_37_ for the same triplet (ΔΔΔ*G*°_37_) was remarkably small, with a maximum of 0.2 kcal mol^−1^ (±0.1 kcal mol^−1^). The differences in ΔΔ*H*° and −*T*ΔΔ*S°* at 37 °C (ΔΔΔ*H*° and −*T*ΔΔΔ*S*°) for the same triplet were larger than those for ΔΔ*G*°_37_ (Figure 3). Except for the ii series, the triplets were determined to be enthalpically or entropically stabilized, depending on the specific triplet. In terms of sequence characteristics, those containing TT(L) (iii(3), iii(5), iv(3), and iv(5)) were the most enthalpically stabilized (ΔΔ*H*°: −6.8 to −2.5 kcal mol^−1^), while the AT(L)C triplets (i(3) and i(4)) and one AT(L)G triplet (ii(3)) were the only sequences that showed entropic stabilization.

## 3. Discussion

### 3.1. ΔG°, ΔH°, and ΔS° Are More Relevant than T_m_ to Evaluate the Thermodynamic Stability of Nucleic Acid Double-Strands Containing a Single LNA Residue

In this study, we evaluated the thermodynamic stability of DNA/RNA duplexes containing a single LNA modification. The stabilities of LNA-containing duplexes have been evaluated in several laboratories using the *T*_m_ or the thermodynamic parameters Δ*G*°, Δ*H*°, and Δ*S*°. Singh et al. determined the *T*_m_ of duplexes formed by LNA-containing DNA and target DNA or RNA [5]. Kaur et al. measured the *T*_m_ of duplexes formed by LNA-containing DNA and complementary DNA or RNA [23,24]. Both studies showed that LNA introduction increased the *T*_m_ of the duplexes. Thus, *T*_m_ values are often used as indicators of binding affinity between antisense oligonucleotides and target sequences. Although *T*_m_ is useful for a rough comparison of duplex stability, care should be taken in handling this value because it depends on the concentration and solution environment. As expressed in Equation (11) (see Section 4.3) and Equation (12), the *T*_m_ depends on the concentration, and the concentration dependence further varies with Δ*H*° and Δ*S*°.

*T*_m_ = Δ*H*°/{Δ*S*° − *R*ln(4/*C*_t_)} (1)

According to Equation (11), the slope of the *T*_m_^−1^ vs. ln(*C*_t_/4) plot is given by *R*/Δ*H*°. Thus, a smaller negative Δ*H*° results in a more negative slope, indicating a higher concentration dependency of *T*_m_. When comparing two duplexes with different Δ*H*° values, the relative magnitude of their *T*_m_ values may reverse depending on the concentration. Therefore, when evaluating the binding affinity of various antisense oligonucleotides, a high *T*_m_ does not necessarily guarantee the functionality of the antisense oligonucleotide at 37 °C.

We propose using Δ*G*°_37_ instead of *T*_m_ in future evaluations of the stabilizing effect of modified nucleic acids for the following reasons. The Δ*T*_m_ per modification is often used to compare the stabilizing effects of modified nucleic acids. If the *T*_m_ increase per modification is the same for antisense oligonucleotides with a single and multiple LNA substitutions, then the following equation would hold:

*T*_m_ (single LNA) − *T*_m_ (no LNA) = *T*_m_ (two LNAs) − *T*_m_ (single LNA).

That is,

2 × *T*_m_ (single LNA) = *T*_m_ (two LNAs) + *T*_m_ (no LNA).

However, suppose this equation is incorrect. Substituting Equation (1) into the above equation yields the following expression:

(supposed false) 2× [Δ*H*°(single LNA)/{Δ*S*°(single LNA) − *R*ln(4/*C*_t_)}] = [Δ*H*°(two LNAs)/{Δ*S*°(two LNAs) − *R*ln(4/*C*_t_)}] + [Δ*H*°(no LNA)/{Δ*S*°(no LNAs) − *R*ln(4/*C*_t_)}] (2)

If a and b represent the changes in Δ*H*° and Δ*S*°, respectively, due to the introduction of one LNA, then Δ*H*° and Δ*S*° for a single LNA and two LNAs can be expressed as follows:

Δ*H*° (single LNA) = Δ*H*° (no LNA) + a (3)

Δ*H*° (two LNAs) = Δ*H*° (no LNA) + 2a (4)

Δ*S*° (single LNA) = Δ*S*° (no LNA) + b (5)

Δ*S*° (two LNAs) = Δ*S*° (no LNA) + 2b (6)

Substituting Equations (3)–(6) into Equation (2), we obtain:

(supposed false) 2× [(Δ*H*°(no LNA) + a)/{Δ*S*° (no LNA) + b − *R*ln(4/*C*_t_)}] = [(Δ*H*°(no LNA) + 2a)/{Δ*S*°(no LNA) +2b − *R*ln(4/*C*_t_)} + Δ*H*° (no LNA)/{Δ*S*° (no LNA) − *R*ln(4/*C*_t_)}] (7)

Equation (7) holds only for b = 0. However, enthalpy and entropy changes are generally compensatory; if one is energetically favorable, then the other is energetically unfavorable. Therefore, there are a few systems that change only the enthalpy, indicating that Equation (7) is not fulfilled in the majority of cases.

However, the thermodynamic stabilization effect can be evaluated using the ΔΔ*G*°_37_ per modification, because the following equation can be applied.

Δ*G*°_37_ (single LNA) − Δ*G*°_37_ (no LNA) = Δ*G*°_37_ (two LNAs) − Δ*G*°_37_ (single LNA), 2Δ*G*°_37_ (single LNA) = Δ*G*°_37_ (two LNAs) + Δ*G*°_37_ (no LNA), (8)

If a and b represent the changes in Δ*H*° and Δ*S*°, respectively, due to the introduction of one LNA, then the following equation can be derived using the relationship Δ*G*° = Δ*H*° − *T*Δ*S*°:

2 × {Δ*H*° (no LNA) + a − *T*(Δ*S*° (no LNA) + b)} = {Δ*H*° (no LNA) + 2a − *T*(Δ*S*° (no LNA) + 2b)}+ Δ*H*° (no LNA) − *T*(Δ*S*° (no LNA)) (9)

This case is valid regardless of the extent to which a and b are thermodynamically affected by each LNA modification. Therefore, it is likely that Δ*G*°, Δ*H*°, and Δ*S*° are more appropriate for evaluating the thermodynamic stabilization effect per LNA modification.

### 3.2. The Thermodynamic Stabilization Mechanism of LNA Substitution for DNA/RNA Duplexes May Be Sequence-Specific

Many reports have clarified the thermodynamic stabilization mechanism of LNA introduction into DNA/DNA [7,21,24,25,26,27,28,29,30,31] and RNA/RNA [22,32,33,34] duplexes, and almost all reports have shown that LNA enhances the thermodynamic stability of the duplexes. However, thermodynamic information on DNA/RNA duplexes containing LNA is scarce [23]. In the development of antisense oligonucleotide therapeutics, thermodynamic parameters that quantify duplex stability under solution conditions similar to the biological conditions at 37 °C are essential for evaluating the binding affinity of drugs. Therefore, we evaluated the thermodynamic properties of LNA containing DNA/RNA duplexes by determining Δ*G*°_37_, Δ*H*°, Δ*S*°, and −*T*Δ*S*°.

An LNA with an immobilized ribose ring was developed to prevent the flexibility of the sugar pucker, which was expected to reduce the entropy loss during duplex formation and entropically increase the binding affinity. However, in several of the sequences used in this study, LNA enthalpically stabilized the duplex when introduced into thymine. Our results suggest that introducing a single LNA in the relatively central region of the antisense DNA leads to unique thermodynamic parameters that vary depending on the sequence. Therefore, the thermodynamic stabilization by LNA may be enthalpy- or entropy-dependent, depending on the sequence.

Other reports have suggested the sequence specificity of stabilization by LNA. Kumar et al. evaluated LNA-containing RNA/RNA duplexes and showed that the introduction of LNA into a base other than cytosine results in enthalpic stabilization due to enhanced stacking interactions, and that the introduction of LNA into an AU base pair with predominant stacking interactions may result in enthalpic stabilization [32]. Additionally, McTigue et al. suggested that the stabilizing effect strongly depends on the DNA sequences of the duplexes [21]. LNA modification of pyrimidine bases showed a greater stabilizing tendency than that of purine bases. Additionally, the stabilizing effect was greater when the base adjacent to the LNA residue was purine, especially when the base was guanine and was enthalpically stabilized. The mechanism of nucleic acid duplex stabilization by LNA is determined by the following four factors: [Entropic factor1] LNA introduction suppresses conformational flexibility and heterogeneity of the sugar pucker to entropically stabilize the nucleic acid duplex via a preorganization effect, as intended in the molecular design. [Entropic factor2] If the structure of single-stranded DNA (including LNA) transforms into the A-form following the introduction of LNA, then the DNA structure in the DNA/RNA duplexes adopts the A-form. This reduces entropy loss during duplex formation owing to the structural change from the B-form to the A-form. [Enthalpic factor1] The introduction of LNA enhances the stacking interactions, resulting in enthalpic stabilization. [Enthalpic factor2] LNA modifications promote the formation of duplexes by facilitating the incorporation of Na^+^, as previously reported, where LNA modifications result in decreased water uptake and increased sodium ion uptake [24]. As discussed above, several factors may contribute to LNA-induced stabilization. Among them, [Enthalpic factor1] is sequence-specific.

### 3.3. The Thermodynamic Stabilization Effect Brought by LNA-T Introduction Should Be Sequence-Dependent

To verify whether the stabilizing effect of LNA-T is sequence-dependent, it is necessary to assess if the results obtained in this study can predict the thermodynamic parameters (Δ*H*°, Δ*S*°, and Δ*G*°_37_) from the NN model. For natural nucleic acid duplexes, Δ*H*°, Δ*S*°, and Δ*G*°_37_ can be predicted from their sequences using the NN parameters. The accurate parameters (Δ*H*°, Δ*S*°, and Δ*G*°_37_) for natural DNA/DNA, RNA/RNA, and DNA/RNA duplexes can be predicted. We hypothesized that the binding affinity of LNA-containing DNA/RNA duplexes could be predicted from the sequence if the parameters for the LNA-containing NN set could be obtained. Based on this concept, Owczarzy et al. calculated novel NN parameters for LNA-containing DNA/DNA duplexes [25]. The present results, although obtained from a limited number of sequences, indicate that the thermodynamic stabilization effect of LNA can be predicted from the sequence alone, even for DNA/RNA duplexes. Δ*G*°_37_, in particular, can be predicted with high accuracy. On the other hand, there are relatively large differences observed in ii(3) and ii(4) for AT(L)G, iii(3) and iii(5) for TT(L)C, and iv(3) and iv(5) for TT(L)A. These pairs, which show significant differences, can all be denoted as WT(L)N (W: A or T). The WT(L)N triplet is expected to be relatively flexible, especially on the 5′ side, where stacking interactions may predominate over hydrogen bonding. In contrast, sequences with AT base pairs on the 3′ side, such as GT(L)T and CT(L)T, are considered relatively rigid, based on the report by Petersen et al. [35]. Flexible duplex structures can partially change their conformation when interacting with a solvent. Such conformational changes could affect the strength of the interaction. If ∆*H*° becomes unstable, then the overall stability would not necessarily change, as the compensation effect would stabilize −*T*∆*S*°, as excellently reported by McTigue et al. in 2004. Similar phenomena were observed for LNA-containing DNA/DNA duplexes [21]. The paper reported that the ∆∆*H*°, ∆∆*S*°, and ∆∆*G*° of the AT(L)G-containing sequences L10d and LT8c were 1.6 and −3.8 kcal mol^−1^, 9.0 and −9.0 cal mol^−1^ K^−1^, and −1.16 and −1.04 kcal mol^−1^, respectively, demonstrating differences in parameters for the identical sequences. Thus, the stability of LNA-containing DNA/RNA duplexes may be related not only to the sequence, as in natural-type DNA/RNA duplexes, but also to other factors that may influence stability, suggesting that correction terms beyond the sequence may be necessary for prediction using the NN parameters. Therefore, further extensive data are required to accurately predict Δ*H*° and Δ*S*°.

For DNA/RNA duplexes, the stabilizing effect (ΔΔ*G*°_37_) obtained when substituting a single thymidine in a sequence with LNA ranged from −2.1 to −1.1 kcal mol^−1^ (average: −1.5 kcal mol^−1^) (Table 1). The ΔΔ*G*°_37_ of sequences with the same combination of bases adjacent to the LNA-modified thymidine showed remarkably close values within an error margin of 0.3 kcal/mol, which is 2–3% of Δ*G*°_37_. Similarly, ΔΔ*H*° and −*T*ΔΔ*S*° also showed close values (Table 1). These results suggest that thermodynamic parameters may have sequence-specific values when the overall base compositions of the oligonucleotides are the same. The small error of ΔΔ*G*°_37_ in this study also suggests that the total stability of DNA/RNA duplexes with an LNA may be correctly predicted by NN parameters with a 3% error margin.

**Figure 4:**
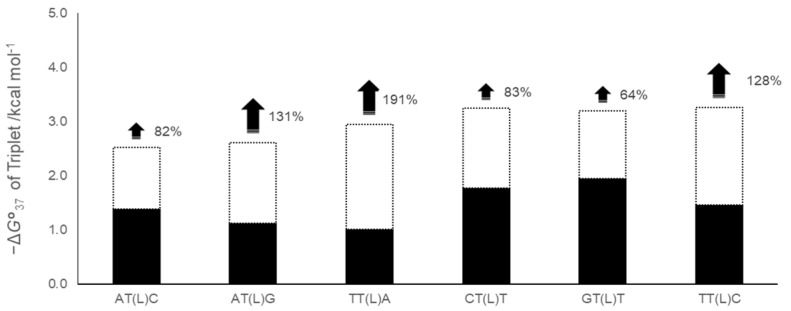
Predicted Δ*G*°_37_ of the triplets including LNA substitution. Δ*G*°_37_ of the triplets obtained in this study are listed in decreasing order. The black bar indicates the predicted Δ*G*°_37_ of the natural-type DNA/RNA duplex in the presence of 100 mM NaCl, obtained by multiplying the Δ*G*°_37_ of the triplet predicted from the NN parameters in the presence of 1 M NaCl by 0.63 (according to Nakano S. et al. [37]). The white bars are the ΔΔ*G*°_37_ obtained in this study.

The thermodynamic stabilization effect (ΔΔ*G*°_37_) of the LNA-containing DNA/RNA duplexes with the same LNA-containing triplet was averaged. The mean values for AT(L)C, AT(L)G, TT(L)A, CT(L)T, GT(L)T, and TT(L)C were 1.1, 1.5, 1.9, 1.5, 1.2, and 1.8 kcal mol^−1^, respectively. To explore potential correlations with the stability of the natural-type triplet, we calculated Δ*G*°_37_ (natural-type DNA/RNA duplex) in a 100 mM NaCl solution, based on the NN parameter of a natural-type DNA/RNA duplex [20,36]. The calculated Δ*G*°_37_ values for the natural triplets ATC, ATG, TTA, CTT, GTT, and TTC were 1.4, 1.1, 1.0, 1.8, 1.9, and 1.5 kcal mol^−1^, respectively. Figure 4 shows the averaged ΔΔ*G*°_37_ of the triplets (white bars) and the Δ*G*°_37_ of the natural-type DNA/RNA triplets (black bars). To assess the influence of the LNA substitution on stabilization, the ratio of ΔΔ*G*°_37_ to Δ*G*°_37_ (natural-type DNA/RNA duplex) was determined (hereafter referred to as the stabilization efficiency) using the following equation:

{ΔΔ*G*°_37_(N_1_N_2_(L)N_3_)/Δ*G*°_37_ (natural-type N_1_N_2_N_3_)} × 100%. (10)

In the natural duplexes, the stability of the TTA triplet was −1.0 kcal mol^−1^, the lowest of the six, whereas the stabilization effect of the LNA stabilized it by 1.9 kcal mol^−1^, the highest of the six. The stabilization efficiency was calculated to be 191%. The natural TTA triplet was less stable than the ATG and ATC triplets; however, the stability of the TTA triplet with LNA was slightly higher than that of the ATG and ATC LNA-containing triplets. In contrast, the GTT and CTT triplets, which were stable in natural duplexes, were affected by the introduction of LNA by 1.2–1.3 and 1.4–1.6 kcal mol^−1^, respectively. These values indicated relatively low stabilization efficiencies of 64% and 83%, respectively. The stability of the original triplets and the stabilization efficiency appear to be independent.

Duplexes with a purine base at the 3′ side exhibited a higher stabilizing effect (191% and 131% for TT(L)A and AT(L)G, respectively), whereas those with a pyrimidine base at the 3′ side exhibited a lower stabilizing effect (64–83% for GT(L)T, AT(L)C, and CT(L)T). However, the stabilizing effect tended to be higher when a thymine base was present on the 5′ side, a trend different from that observed by McTigue et al. For example, TT(L)C, which has a pyrimidine base on the 3′ side, exhibited a higher stabilizing effect than AT(L)C (AT(L)C: 82% < TT(L)C: 128%). Additionally, TT(L)A with a purine base on the 3′ side and a thymine base on the 5′ side exhibited a 191% increase in the stabilizing effect. These results indicate that the combination of base sequences determines the stabilizing effect of LNA.

## 4. Materials and Methods

### 4.1. Oligonucleotide Synthesis

Oligonucleotides were purchased from Gene Design, Inc. (Osaka, Japan). Antisense DNAs with an LNA modification at a thymine nucleoside of i(DNA), ii(DNA), iii(DNA), and iv(DNA) are specified in Table 2. The concentrations of all oligonucleotides were determined from the molar extinction coefficient (ε) estimated from the predicted values of DNA [38].

All measurements were performed in 10 mM neutral phosphate buffer (1 mM EDTA, 100 mM NaCl, pH 7.0) with a 1:1 ratio of antisense DNA and complementary RNA. The samples were heated to 95 °C and subsequently cooled to 10 °C at a rate of 0.5 °C/min to form duplexes before analysis.

### 4.2. Circular Dichroism

To confirm the two-state transition of duplex denaturation, the temperature dependence of circular dichroism (CD) spectra for i(DNA/RNA), ii(DNA/RNA), iii(DNA/RNA), and iv(DNA/RNA) was obtained using a J-820 spectrometer (JASCO, Tokyo, Japan) (Figure 1). The oligonucleotides (total concentration (*C*_t_): 30 μM) were heated from 20 to 60 °C, and spectra were measured at 5 °C intervals. The temperatures of the samples were controlled using a JASCO PTC-423L instrument. All spectra were recorded between 200 and 340 nm, with an average of six scans.

### 4.3. Melting Experiments

UV melting experiments were performed to measure the temperature dependence of the absorbance at 260 nm on a UV-1800 using the TMSPC-8 temperature controller (Shimadzu, Kyoto, Japan). UV measurements were performed using quartz cuvettes with path lengths of 1 and 0.1 cm. A pressure bond adhesive seal on the quartz cuvettes was used to prevent evaporation. The melting and annealing curves were obtained by heating or cooling the samples at a rate of 0.5 °C min^−1^. The obtained melting curves were analyzed using Equation (11), where *R* denotes the gas constant, to calculate Δ*G*°_37_, Δ*H*°, Δ*S*°, and *T*_m_. Hereafter, this analysis method is referred to as curve fitting.

ln*K* = −Δ*H*°/*RT* + Δ*S*°/*R* (11)

Additionally, the correlation between *T*_m_ and *C*_t_ of the oligonucleotides was analyzed using Equation (12) to calculate Δ*H*° and Δ*S*°, and then Δ*G*°_37_ (Figures S1–S4). Hereafter, this analysis method is referred to as the log*C*_t_ plot.

1/*T*_m_ = ln (*C*_t_/4) × *R*/Δ*H*° + Δ*S*°/Δ*H*° (12)

The averages of the parameters obtained from the two methods, curve fitting and the log*C*_t_ plot, were used for data calculation (Table 1).

### 4.4. Calculation of the Stabilization Effect of LNA

The differences in thermodynamic parameters brought by LNA introduction were evaluated as ΔΔ*G*°_37_, ΔΔ*H*°, and ΔΔ*S*° (Table 1), using the following equations:

ΔΔ*G*°_37_ = Δ*G*°_37_ (LNA/RNA) − Δ*G*°_37_ (DNA/RNA) (13)

ΔΔ*H*° = Δ*H*° (LNA/RNA) − Δ*H*° (DNA/RNA) (14)

ΔΔS° = Δ*S*° (LNA/RNA) − Δ*S*° (DNA/RNA) (15)

## 5. Conclusions

In conclusion, the stabilization mechanism and effect are determined by the sequence. To the best of our knowledge, this is the first study to verify sequence specificity of the thermodynamic effects of DNA/RNA duplexes brought about by the introduction of a single LNA-T residue. Our study suggests that the stabilizing effect of LNA-T on DNA and RNA is unique and depends on the combination of adjacent bases. We propose a hypothesis that the phenomena observed with LNA-thymidine may be extended to the other three LNA nucleosides. Thus, the thermodynamic stability of LNA-containing antisense DNA and RNA duplexes can be predicted based on this sequence. These results provide meaningful guidance for the future design of LNA-modified antisense nucleic acids.

## Figures and Tables

**Table 1:** Thermodynamic parameters for duplex formation and differences due to LNA introduction.

Oligo Name	Triplet Including LNA	Δ*G*°_37_	Δ*H*°	Δ*S*°	−*T*Δ*S*° *	*T*_m_ **	ΔΔ*G*°_37_	ΔΔ*H°*	−*T*ΔΔ*S*° *	Δ*T*_m_ **
		(kcal mol^−1^)	(kcal mol^−1^)	(cal mol^−1^ K^−1^)	(kcal mol^−1^)	(°C)	(kcal mol^−1^)	(kcal mol^−1^)	(kcal mol^−1^)	(°C)
i(DNA)		−8.5	−85.2	−247.5	76.8	41.4				
i(3)	AT(L)C	−9.7	−84.0	−239.6	74.3	46.4	−1.2	1.2	−2.4	4.9
i(4)	AT(L)C	−9.6	−84.1	−240.2	74.5	46.1	−1.1	1.2	−2.3	4.6
ii(DNA)		−7.3	−77.7	−227.0	70.4	37.2				
ii(3)	AT(L)G	−8.9	−77.6	−221.5	68.7	43.3	−1.5	0.2	−1.7	6.1
ii(4)	AT(L)G	−8.8	−79.5	−228.2	70.8	42.8	−1.4	−1.8	0.4	5.6
iii(DNA)		−12.1	−89.9	−250.8	77.8	54.7				
iii(2)	GT(L)T	−13.5	−91.8	−252.4	78.3	59.3	−1.3	−1.9	0.5	4.7
iii(3)	TT(L)C	−14.1	−96.7	−266.4	82.6	60.4	−1.9	−6.8	4.8	5.7
iii(4)	GT(L)T	−13.3	−91.8	−253.2	78.5	58.6	−1.2	−1.9	0.8	3.9
iii(5)	TT(L)C	−13.8	−94.3	−259.6	80.5	60.0	−1.7	−4.4	2.8	5.3
iv(DNA)		−8.4	−88.6	−258.7	80.2	40.7				
iv(2)	CT(L)T	−10.0	−92.5	−266.1	82.5	46.3	−1.6	−3.9	2.3	5.5
iv(3)	TT(L)A	−10.5	−95.1	−272.7	84.6	47.6	−2.1	−6.4	4.4	6.9
iv(4)	CT(L)T	−9.8	−91.6	−263.6	81.8	45.7	−1.4	−2.9	1.5	4.9
iv(5)	TT(L)A	−10.2	−91.0	−260.5	80.8	47.2	−1.8	−2.4	0.5	6.4

* −*T*ΔS° values are calculated at 37 °C. ** *T*_m_ indicates the value at *C*_t_ = 30 μM. Errors for Δ*G*°_37_, Δ*H*°, Δ*S*°, and −*T*Δ*S*° were ±0.0 to 0.2 kcal mol^−1^, ±1.4 to 7.0 kcal mol^−1^, ±4.5 to 20.2 cal mol^−1^ K^−1^, and ±1.4 to 6.3 kcal mol^−1^, respectively. There are some discrepancies between the ΔΔ and Δ values in Table 1 because the ΔΔ values were calculated using Δ values rounded to two decimal places. For example, ΔΔ*G*°_37_ (ii(3)) = Δ*G*°_37_ (ii(3)) − Δ*G*°_37_ (ii(DNA)) = −8.87 − (−7.34) = −1.53 (−1.5 kcal/mol).

**Table 2:** List of sequences used in the study.

Oligo Name	Sequence (5′ to 3′)
i(RNA)	AAUGAUGAUGAA
i(DNA)	TTCATCATCATT
i(3)	TTCAT(L)CATCATT
i(4)	TTCATCAT(L)CATT
ii(RNA)	AAUCAUCAUCAA
ii(DNA)	TTGATGATGATT
ii(3)	TTGAT(L)GATGATT
ii(4)	TTGATGAT(L)GATT
iii(RNA)	ACGAACGAACGA
iii(DNA)	TCGTTCGTTCGT
iii(2)	TCGT(L)TCGTTCGT
iii(3)	TCGTT(L)CGTTCGT
iii(4)	TCGTTCGT(L)TCGT
iii(5)	TCGTTCGTT(L)CGT
iv(RNA)	AGUAAGUAAGUA
iv(DNA)	TACTTACTTACT
iv(2)	TACT(L)TACTTACT
iv(3)	TACTT(L)ACTTACT
iv(4)	TACTTACT(L)TACT
iv(5)	TACTTACTT(L)ACT

LNA residues are indicated by (L) on the right (3′) side of the respective base symbol. LNA residues and the nearest neighbor nucleotides are underlined. The last number of the oligo names indicates the LNA position, e.g., i(3) indicates that the third thymine from the 5′ end of i(DNA) is substituted by LNA.

## Data Availability

Data is contained within the article and Supplementary Materials.

## Supplementary Materials

The following supporting information can be downloaded at: https://www.mdpi.com/article/10.3390/ijms252413240/s1.
