# Supplementary material for: Sequence-Specific Free Energy Changes in DNA/RNA Induced by a Single LNA-T Modification in Antisense Oligonucleotides
Source: Int J Mol Sci. 2024 Dec 10;25(24):13240. doi: 10.3390/ijms252413240 (PMC11676002; doi:10.3390/ijms252413240)
Supplement: Supplementary file 1 [file ijms-25-13240-s001.zip › ijms-3309504-supplementary.pdf]

Supplement Equations

The presence of "4" in eq.1 arises from the derivation based on a system where duplexes form from non-self-complementary single strands, as described below.

$ss_1+ss_2 \rightleftarrows ds$ (ss: single strands, ds: duplexes).

The binding constant K is defined as:

$K=[ds]_{eq}/[ss_1]_{eq}[ss_2]_{eq}$ ("eq" means equilibrium)

Since the single chains are in the same ratio, the initial concentration of each can be expressed using C_t:

$[ss_1]_0=[ss_2]_0=C_t/2$

Here, α is defined as the ratio of nucleic acids that form duplexes at a given temperature. The respective concentrations are expressed as follows;

$[ss_1]=[ss_2]=\{C_t\,(1-\alpha)\}/2,$

$[ds]=(C_t\,\alpha)/2$

Substituting into the expression for K, we obtain:

$K=(C_t\,\alpha)/2/[\{C_t\,(1-\alpha)\}/2]2$

When $T=T_m$, $\alpha=1/2$, $K=C_t/4$. Substituting these into equation eq.11 $\ln K=-\Delta H°/RT+\Delta S°/R$, we get $\ln(4/C_t)=-\Delta H°/RT_m+\Delta S°/R$. Transforming this, we obtain eq.1 $T_m = \Delta H° / \{\Delta S° - R\ln(4/C_t)\}$.

Table S1. Averaged thermodynamic parameters obtained by curve fitting and logCt plot.

**oligo name**	**sequence (5′ to 3′)**		**ΔG°_{37} (kcal mol^{-1})**		**ΔH° (kcal mol^{-1})**		**ΔS° (cal mol^{-1} K^{-1})**		**$-T\Delta S^{\circ}$ (kcal mol^{-1})**	
i(DNA)	TTCATCATCATT	curve fitting	-8.5	± 0.1	-86.0	± 2.2	-250.0	± 7.0	77.5	± 2.2
		logC_t plot	-8.5	---------	-84.5	± 5.4	-245.0	± 15.8	76.0	± 4.9
i(3)	TTCAT(L)CATCATT	curve fitting	-9.8	± 0.1	-87.5	± 2.0	-250.4	± 6.3	77.7	± 2.0
		logC_t plot	-9.6	---------	-80.6	± 1.8	-228.8	± 5.2	71.0	± 1.6
i(4)	TTCATCAT(L)CATT	curve fitting	-9.6	± 0.1	-87.2	± 2.8	-250.1	± 9.0	77.6	± 2.8
		logC_t plot	-9.5	---------	-80.9	± 2.8	-230.2	± 8.1	71.4	± 2.5
ii(DNA)	TTGATGATGATT	curve fitting	-7.3	± 0.0	-78.0	± 4.1	-227.9	± 13.2	70.7	± 4.1
		logC_t plot	-7.3	---------	-77.5	± 2.6	-226.1	± 7.7	70.1	± 2.4
ii(3)	TTGAT(L)GATGATT	curve fitting	-8.9	± 0.0	-77.9	± 1.6	-222.7	± 5.3	69.1	± 1.6
		logC_t plot	-8.9	---------	-77.2	± 3.6	-220.4	± 10.3	68.4	± 3.2
ii(4)	TTGATGAT(L)GATT	curve fitting	-8.8	± 0.0	-79.2	± 1.6	-227.1	± 5.1	70.4	± 1.6
		logC_t plot	-8.8	---------	-79.9	± 2.1	-229.3	± 6.2	71.1	± 1.9
iii(DNA)	TCGTTCGTTCGT	curve fitting	-12.2	± 0.2	-90.9	± 3.8	-254.0	± 11.6	78.8	± 3.6
		logC_t plot	-12.1	---------	-88.9	± 5.6	-247.6	± 15.6	76.8	± 4.8
iii(2)	TCGT(L)TCGTTCGT	curve fitting	-13.6	± 0.1	-93.1	± 1.7	-256.4	± 5.3	79.5	± 1.6
		logC_t plot	-13.4	---------	-90.5	± 5.5	-248.5	± 15.2	77.1	± 4.7
iii(3)	TCGTT(L)CGTTCGT	curve fitting	-13.9	± 0.1	-94.4	± 2.0	-259.4	± 6.2	80.4	± 1.9
		logC_t plot	-14.2	---------	-99.0	± 5.7	-273.4	± 15.8	84.8	± 4.9
iii(4)	TCGTTCGT(L)TCGT	curve fitting	-13.4	± 0.1	-92.6	± 1.4	-255.6	± 4.5	79.3	± 1.4
		logC_t plot	-13.2	---------	-91.0	± 6.6	-250.8	± 18.2	77.8	± 5.6
iii(5)	TCGTTCGTT(L)CGT	curve fitting	-13.9	± 0.1	-95.2	± 1.7	-262.2	± 5.2	81.3	± 1.6
		logC_t plot	-13.8	---------	-93.5	± 5.8	-257.1	± 16.1	79.7	± 5.0
iv(DNA)	TACTTACTTACT	curve fitting	-8.4	± 0.1	-87.3	± 2.1	-254.3	± 6.7	78.9	± 2.1
		logC_t plot	-8.4	---------	-90.0	± 3.5	-263.1	± 10.3	81.6	± 3.2
iv(2)	TACT(L)TACTTACT	curve fitting	-9.9	± 0.1	-87.9	± 3.1	-251.5	± 9.8	78.0	± 3.0
		logC_t plot	-10.1	---------	-97.1	± 2.3	-280.6	± 6.6	87.0	± 2.0
iv(3)	TACTT(L)ACTTACT	curve fitting	-10.3	± 0.1	-89.2	± 3.2	-254.1	± 10.3	78.8	± 3.2
		logC_t plot	-10.6	---------	-101.0	± 2.5	-291.4	± 7.1	90.4	± 2.2
iv(4)	TACTTACT(L)TACT	curve fitting	-9.8	± 0.1	-89.5	± 3.7	-256.9	± 11.8	79.7	± 3.7
		logC_t plot	-9.8	---------	-93.7	± 5.6	-270.3	± 16.3	83.8	± 5.1
iv(5)	TACTTACTT(L)ACT	curve fitting	-10.2	± 0.1	-89.6	± 2.0	-255.9	± 6.5	79.4	± 2.0
		logC_t plot	-10.3	---------	-92.5	± 7.0	-265.0	± 20.2	82.2	± 6.3
