# Supplementary figures and images for: Sequence-Specific Free Energy Changes in DNA/RNA Induced by a Single LNA-T Modification in Antisense Oligonucleotides
Source: Int J Mol Sci. 2024 Dec 10;25(24):13240. doi: 10.3390/ijms252413240 (PMC11676002; doi:10.3390/ijms252413240)

## Slide 1
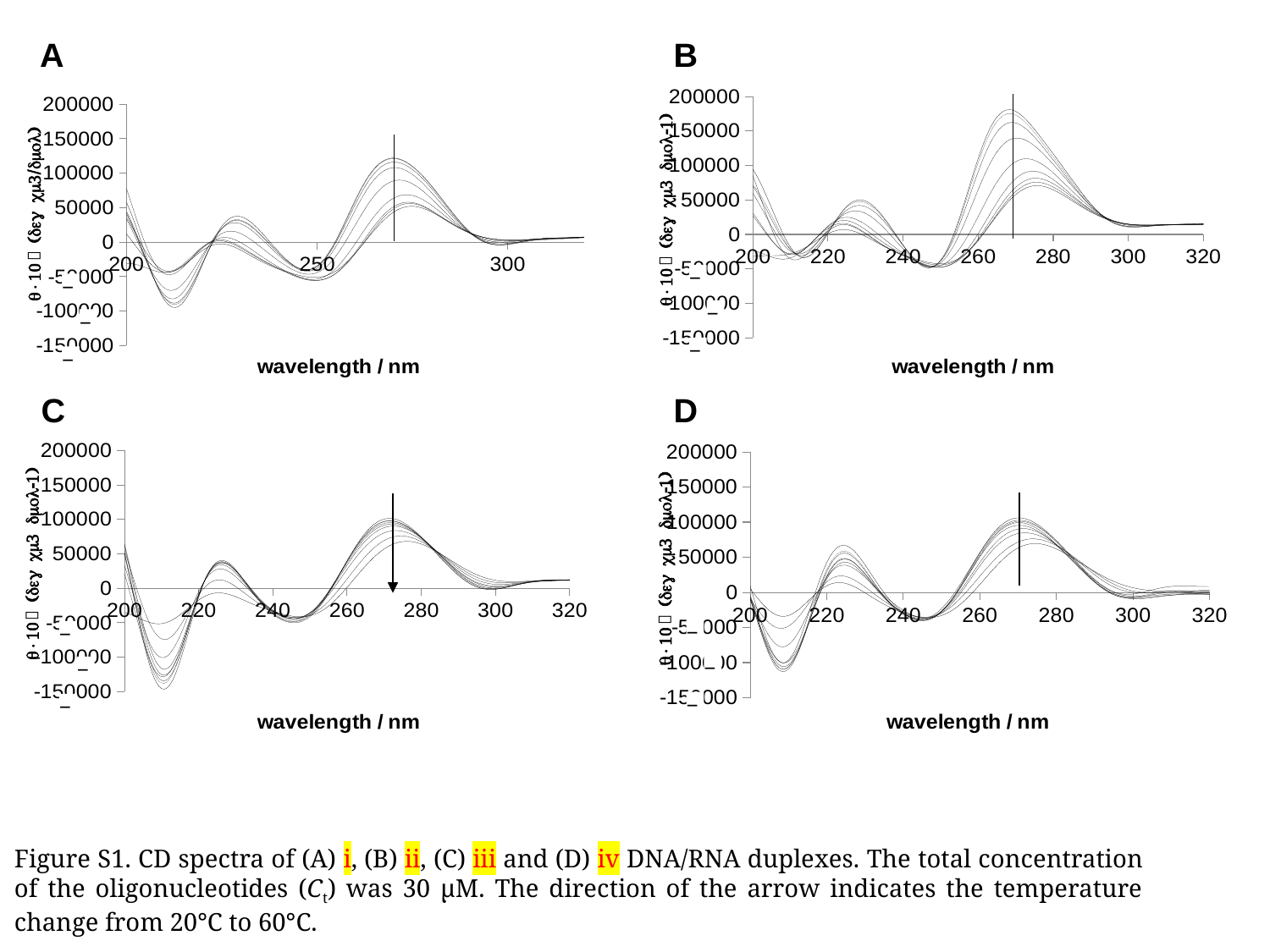

## Slide 2
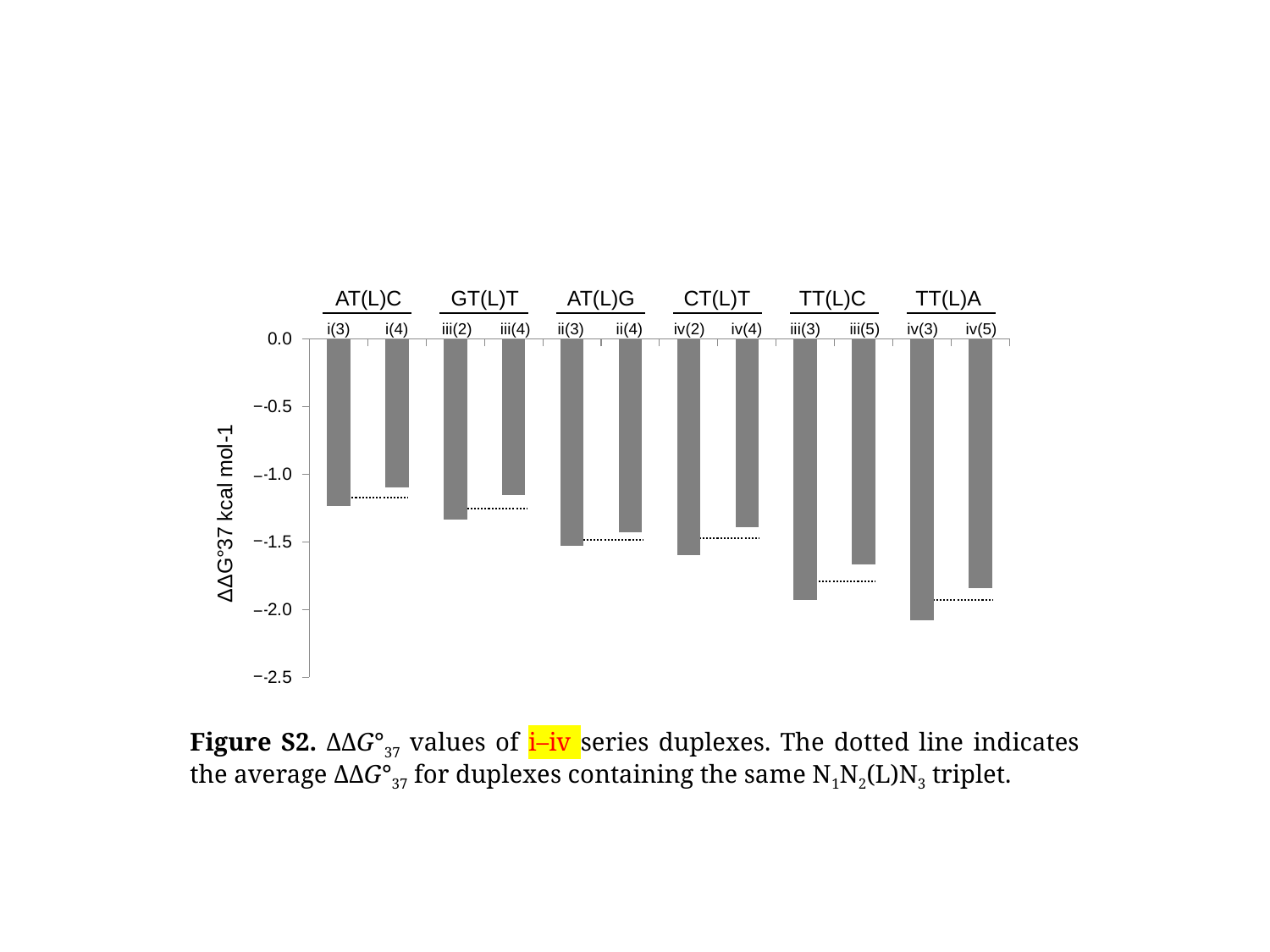

## Slide 3
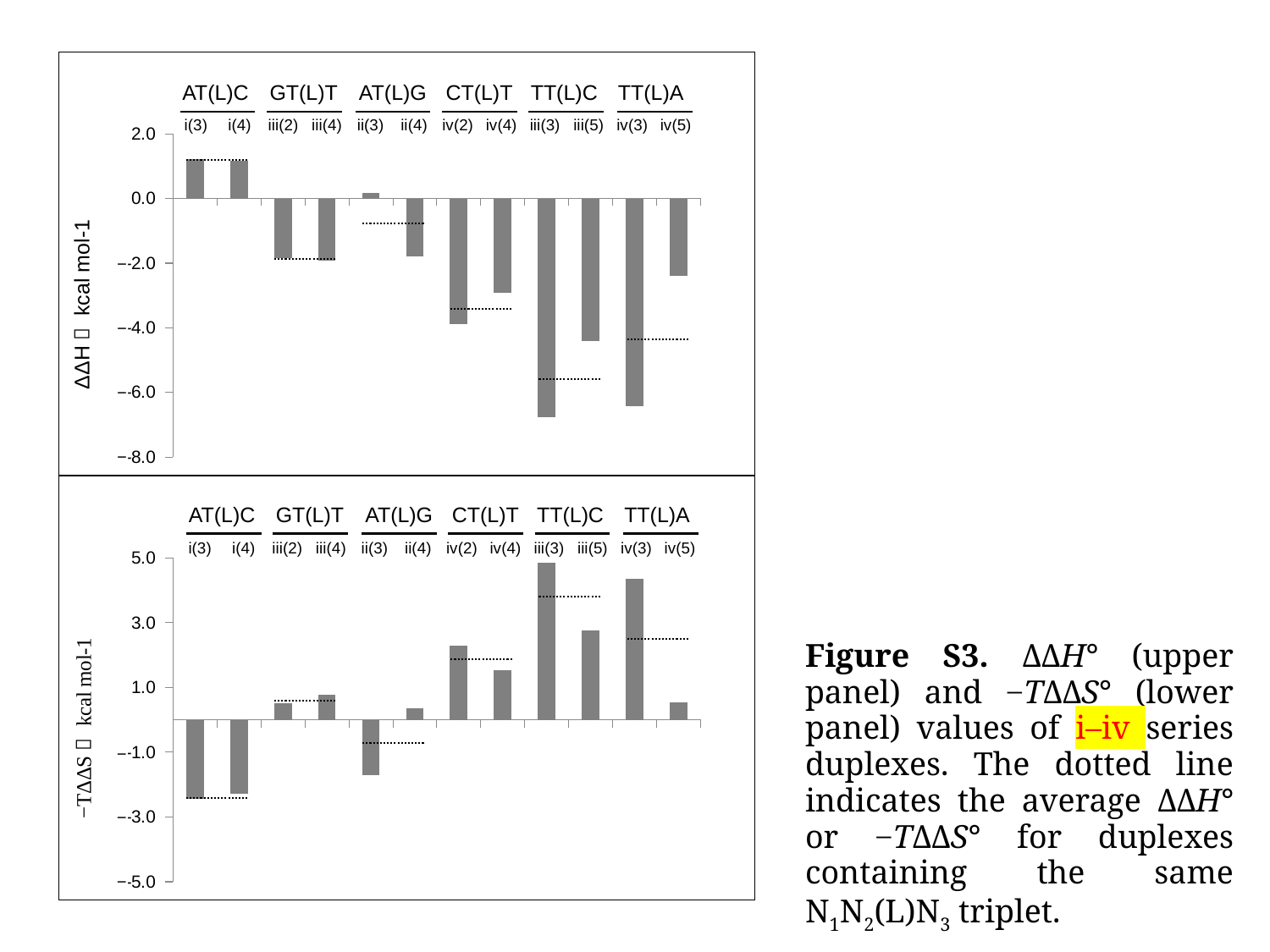

## Slide 4
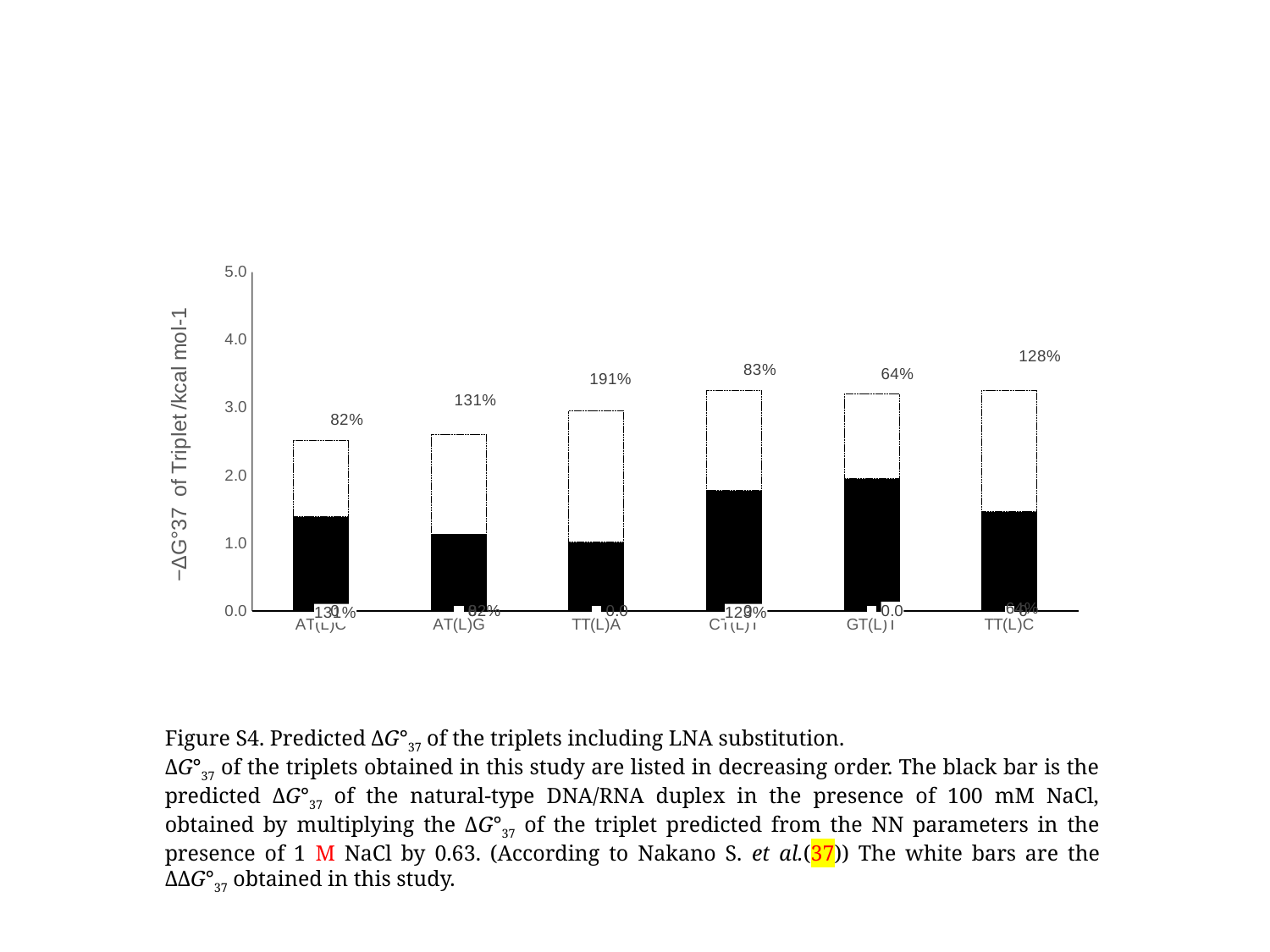

Supplement: Supplementary file 1 [file ijms-25-13240-s001.zip › ijms-3309504-supplementary.pptx]
